# Effects of a self‐management training for people with intellectual disabilities

**DOI:** 10.1111/jar.12536

**Published:** 2018-10-23

**Authors:** Janice Sandjojo, Aglaia M. E. E. Zedlitz, Winifred A. Gebhardt, Joop Hoekman, Jeanet A. den Haan, Andrea W. M. Evers

**Affiliations:** ^1^ Health, Medical and Neuropsychology Unit Leiden University Leiden The Netherlands; ^2^ Leiden Institute for Brain and Cognition (LIBC) Leiden University Leiden The Netherlands; ^3^ Raamwerk Noordwijkerhout The Netherlands; ^4^ Clinical Child and Adolescent Studies Leiden University Leiden The Netherlands; ^5^ Department of Psychiatry Leiden University Medical Center Leiden The Netherlands

**Keywords:** daily living skills, independence, intellectual disabilities, personal goal attainment, self‐management, training

## Abstract

**Background:**

To help people with intellectual disabilities lead a more independent life, it is important to promote their self‐management. This study evaluated the effectiveness of a self‐management training for people with intellectual disabilities directed at independent functioning in daily life.

**Method:**

In the training, 17 people with intellectual disabilities worked on personal self‐management goals covering a wide range of everyday affairs. Primary outcome measures focused on goal attainment, independence and support needs. Moreover, outcomes regarding psychopathological behaviour and quality of life were explored. Data were collected before and at the start of the training, and 3, 6, 9 and 12 months later.

**Results:**

The training contributed to the attainment of self‐management goals and to the reduction in support needs (*p *<* *0.01). There were no changes in independence, psychopathological behaviour and quality of life.

**Conclusions:**

Results indicate that the training supports people with intellectual disabilities to self‐manage their daily affairs.

## 1 INTRODUCTION

Intellectual disabilities are commonly operationalised as deficits in intellectual and adaptive functioning, as measured by standardized tests (e.g., IQ score < 70). The disabilities have an onset during the developmental period (e.g., <18 years) and lead to limitations in daily life for which ongoing support is needed. These deficits affect not only independent functioning at home, but also participation in social, community, academic or occupational activities (American Psychiatric Association, 2013; Schalock et al., 2010). The United Nations (2006) have declared that people with intellectual disabilities should be enabled to live as independently as possible, which is also desired by many individuals with intellectual disabilities (Bond & Hurst, 2010; Haigh et al., 2013; Kuijken, Naaldenberg, Nijhuis‐van der Sanden, Schrojenstein‐Lantman, & de Valk, 2016). Increasing their abilities to independently manage their affairs could enhance their quality of life and community participation (Dollar, Fredrick, Alberto, & Luke, 2012; Sigafoos et al., 2005). Furthermore, it could reduce behavioural problems (García‐Villamisar, Dattilo, & Matson, 2013) and the need for support from professionals and family members who now often feel overburdened (Dawson et al., 2016; Hermsen, Embregts, Hendriks, & Frielink, 2014; Vilaseca et al., 2017). Therefore, interventions are required that promote self‐management of people with intellectual disabilities.

Self‐management is an overarching term involving all cognitions and actions of a person that deliberately influence his or her behaviour in order to realize self‐selected outcomes (Browder & Shapiro, 1985). Self‐management thus includes the autonomy to self‐determine one's choices to lead one's life according to one's own preferences (Tonkens & Weijers, 1999; Wehmeyer & Abery, 2013; Wehmeyer, Kelchner, & Richards, 1996). Furthermore, self‐management involves independence and self‐reliance, which encompass the abilities to take actions to manage one's affairs and to provide for oneself, thereby relying on one's own efforts, resources, judgement and abilities (Sandjojo et al., 2018).

Various studies on promoting self‐management in people with intellectual disabilities have been conducted. However, most of these studies have included only a very small sample and have investigated only one type of approach (e.g., self‐instruction) or a singular domain (e.g., grocery shopping). To our best knowledge, none of the investigated interventions were generalized to a wider range of people with intellectual disabilities with different self‐management goals. Previous research focused, for example, on prompting (Bouck, Satsangi, & Bartlett, 2017; Dollar et al., 2012; Wu, Wheaton, & Cannella‐Malone, 2016), the use of technology (Cannella‐Malone et al., 2006; Cavkaytar, Acungil, & Tomris, 2017; Cullen, Alber‐Morgan, Simmons‐Reed, & Izzo, 2017; Douglas, Ayres, & Langone, 2015; Ramdoss et al., 2012), employment (Gilson, Carter, & Biggs, 2017; Gomes‐Machado, Santos, Schoen, & Chiari, 2016) or health behaviour (Taggart et al., 2015; Wilson & Goodman, 2011). Most studies presented promising results, but drawing firm conclusions about the effects of self‐management interventions is difficult due to studies’ narrow focus and methodological limitations. However, previous studies emphasized that overall, it is important that interventions are (a) tailored to the needs and personal situations of people with intellectual disabilities (Douglas et al., 2015; Goldschmidt & Song, 2017; Hale, Trip, Whitehead, & Conder, 2011; Kuijken et al., 2016; Young, Naji, & Kroll, 2012), (b) that their support network is involved (Hale et al., 2011; Young et al., 2012) and (c) that attention is paid to the transfer of learnt skills to daily life (Gilson et al., 2017; Goldschmidt & Song, 2017).

In this study, for the first time the Academy of Independence (AoI) was evaluated, which is a self‐management training for people with intellectual disabilities that incorporates the above‐mentioned three important elements. This training is tailored to an individual's personal self‐management goal (PSMG) and to their preferences regarding how they would like to work on this PSMG and is not limited to a singular domain, or a specific strategy or approach. Participants determine themselves which goal(s) within the domain of self‐management they want to train, which can concern a daily living skill, but also, for example, dealing with emotions or social situations. This intrinsic motivation can benefit their personal growth (Ryan & Deci, 2000). The training also takes individuals’ abilities and disabilities into account, actively involves the support network and fosters the transfer to daily life. The primary purpose of this study was to investigate whether this self‐management training would support people with intellectual disabilities to reach their PSMGs and whether it would increase their independence and reduce their support needs. Furthermore, the present authors explored whether the training would reduce psychopathological behaviour and enhance quality of life.

## 2 METHOD

### 2.1 Procedure

The Medical Ethics Committee of the Leiden University Medical Center declared that neither formal medical ethical approval nor written informed consent was necessary. Participants were recruited at Raamwerk, a care organization that provides housing and day care services to people with intellectual disabilities in Noordwijkerhout, the Netherlands. Staff of Raamwerk asked their clients if they wanted to take part in the self‐management training. If a client and his or her legal representative agreed on taking part, they received a letter which explained the study and informed them that data would be handled anonymously. If someone would have objected to participate in the study, that participant would have been excluded from the data collection, but not from the training. However, this did not occur. Data were collected 6 months before the start of the training (T0), at the start of the training (T1), and 3, 6, 9 and 12 months later (T2–T5). Before the start of the training (i.e., in the 6 months between T0 and T1), participants received care as usual.

### 2.2 Participants

Participants had to be adults (≥18 years) diagnosed with intellectual disabilities, and they at least had to be able to focus on the instructions and exercises during the training. The latter was based on the clinical judgement of someone's support staff. If someone's personal situation would significantly interfere with following the training (e.g., due to severe psychiatric problems), that person was not eligible for participation. There was no minimal level of cognitive functioning required (e.g., regarding language skills or intelligence level). Seventeen people with intellectual disabilities enrolled in the training. Between T1 and T2, one participant moved away and therefore dropped out of the study. Personal information regarding age, gender and type of housing was collected. All participants lived within the compound of Raamwerk in Noordwijkerhout, most participants lived in a group home, but some lived semi‐independently in their own apartment, receiving only ambulatory support. Diagnostic information was obtained from their electronic client records. Comorbid conditions as classified in the Diagnostic and Statistical Manual of Mental Disorders (American Psychiatric Association, 2013) were common (e.g., autism spectrum disorders and attention‐deficit hyperactivity disorder). Descriptive statistics of the participants are presented in Table 1.

**Table 1:** Descriptive statistics of participant groups

	Intervention group (*n *=* *17)
Male, *n (%)*	12 (70.6)
Age in years, M (*SD*)	35.9 (13.9)
Level of intellectual disabilities, *n*	
Borderline	2
Mild	12
Moderate	3
Full Scale IQ, M (*SD*)	61.1 (9.6)
Neuropsychiatric comorbidity, *n*	
Autism spectrum disorder	6
Other	5
Housing	
Group home	13
Semi‐independent	4

### 2.3 Training

The self‐management training is developed and described in greater detail by the Academy of Independence (Academie voor Zelfstandigheid, 2015). The aim of the AoI is to promote independent functioning of people with intellectual disabilities and to enable their equal participation in society. Its approach is founded on the methodology “On Your Own Two Feet” (Sandjojo et al., 2018; Scholten & Schuurman, 2008). The AoI's core values encompass a positive and respectful approach, effective learning, nourishment of self‐worth and creating a sense of responsibility in its trainees. The AoI specifically focuses on the abilities and talents of people with intellectual disabilities, instead of their disabilities. AoI trainers encourage and coach people with intellectual disabilities to think and handle things themselves, instead of taking over from them, thereby facilitating their development and sense of responsibility.

The training was implemented within day care services. Experienced support staff received a 3‐day training to become AoI trainers. On average, there were two trainers guiding four participants. At the start of each participant's training, trainers used motivational interviewing techniques (Miller, 1996) to discover the PSMGs. Participants were free to choose the number of PSMGs they wanted to work on targeting a skill needed at home, at work or in their leisure time. Goals had to be specific, measurable, achievable and relevant. Examples of our participants’ PSMGs included cleaning the bathroom, cycling to work and using the Internet. Participants were trained for about half a day per week per PSMG. Training modules took a step‐by‐step approach and used easy‐to‐understand language and many photographs and pictograms. Trainers ensured that each participant's training was tailored to their abilities, disabilities and preferences regarding how they would like to attain their PSMGs, by continuously consulting them about how they would like to be trained. For example, if a participant was unable to read, the training was presented more orally or visually, for example, with demonstrations, role‐play or video material. Another example of this tailored approach is that when a participant would report to have difficulties with remembering the steps of a certain task, the trainer would ask what could help him or her, which could be making a personalized checklist together with self‐selected instructions, photographs and pictograms. Other behaviour change techniques (Michie et al., 2011) that were used by the trainer included prompting, instructions, modelling and giving feedback (praise, corrective and descriptive feedback). Which behaviour change techniques were applied was based on the goals, needs and preferences of a participant at a certain moment, in close consultation with the participant. At the start of each training day, trainers talked with participants about what they had done during the previous training and what they had done the rest of the week with regard to their PSMGs. Participants were also asked how they would like to work on their PSMG that training day. In case participants reported or showed to have difficulties with what was previously trained, instructions and exercises were repeated until the participant was ready for a more advanced step. Then, together with the trainer, the participant worked on acquiring further knowledge and skills necessary for obtaining the PSMG. At the end of a training day, trainers provided participants with a self‐evaluation form on which they could reflect what they had done and learnt that day and also to look ahead at what they would practice outside of the training during the rest of the week and what they would like to do in the following training session. Some participants were able to do this independently, but if a participant could not read or write or reported to have difficulties with this reflection, the trainer helped the participant to fill in the form. In both cases, this self‐evaluation was discussed with the trainer, who also provided feedback. To foster the transfer of learnt skills to daily life, trainers also practised with participants at their home or work locations or in the community. Participants also took their training material home so they could practise in their everyday environment. Trainers also held close contact with involved family members and support staff to ensure that the support network also knew what they could practice with them. Once a participant reached a PSMG and finished the training module, he or she received a certificate and was given the option to start with a new PSMG directed at a different skill or to leave the training.

### 2.4 Data collection and outcome measures

Primary outcome measures regarded the assessment of goal attainment, independence and support needs. Secondary measures concerned psychopathological behaviour and quality of life. The questionnaires INVRA‐Home (*INventarisatie Van RedzaamheidsAspecten*; Inventory of Independence Aspects), INVRA‐Work and the Supports Intensity Scale (SIS) are more time‐consuming. Therefore, these were only collected at T0, T1, T3 and T5. All other questionnaires were filled in at all six measurement points (T0–T5). To limit the burden on participants, they were only involved in the questionnaire on quality of life. For all other measures, the personal tutors from the residential facility and from day care services provided the relevant information.

#### 2.4.1 Attainment of personal self‐management goals (PSMGs)

To evaluate the extent to which participants reached their PSMGs, Goal Attainment Scaling (GAS) was used (Bovend'Eerdt, Botell, & Wade, 2009; Kiresuk & Sherman, 1968; Turner‐Stokes, 2010). GAS can be flexibly applied to different individuals and goals, also in people with intellectual disabilities (Mate‐Kole, Danquah, Twum, & Danquah, 1999). For each PSMG, a five‐point scale was constructed together with the input of the participant and the AoI trainer. Each level of the GAS had to be specific and measurable. The level of the participant before working on a goal was set at −2 (e.g., participant does not know the value of any of the euro coins and bills). The desired PSMG was set at level 0 (e.g., participant knows the value of all euro coins and bills). If the participant had already made progress, but not enough to reach the PSMG, the level was scored as −1 (e.g., participant knows the value of some, but not all euro coins and bills). A score of +1 or +2 could be obtained if (much) more than the expected goal was achieved (e.g., level +1: Participant knows the value all of the euro coins and bills and can put together amounts up to €5; level +2: Participant knows the value all of the euro coins and bills and can put together amounts up to €10). The GAS was scored every 3 months (T1–T5) by evaluating with AoI trainers, participants and sometimes support staff from group homes and day care which GAS level was attained by the participant at that point in time. As the levels were specified beforehand, this could be done easily and objectively.

Participants could start with a new PSMG once they attained their previous PSMG (with a GAS score of 0 or higher). The mean raw GAS score was used for the statistical analyses. In addition, learning curves were analysed visually.

#### 2.4.2 Independence in general

The Social Functioning Scale for the Mentally Retarded‐Plus (SFSMR‐P; Kraijer & Kema, 2004) consists of 63 items addressing several components of independent functioning at home, at work and in social situations. The personal tutors filled in whether a participant currently performed these activities independently (score of 1) or not (score of 0). The mean item score was used for the analyses. The SFSMR‐P is widely used in Dutch care organizations for people with intellectual disabilities, and both the reliability and construct and criterion validity were found to be good (Evers, van Vliet‐Mulder, & Groot, 2000).

#### 2.4.3 Independence at home and in the community

The level of independence at home and in the community was assessed with INVRA‐Home (Douma, Mulder, & Scholten, 2001b). This questionnaire is developed for the field of intellectual disabilities and lists 114 abilities and skills belonging to several aspects of independence: personal care and health, household competence, cognitive competence, societal competence and social interaction. The personal tutor from the residential facility scored whether the participant performed the activities: (a) independently, (b) on his or her own initiative, (c) for the most part and (d) in an acceptable manner. Per item, a score from 0 to 4 could be obtained. The sum of all item scores was used for the analyses. The inter‐rater reliability was found to be substantial, and the internal consistency is high (Douma et al., 2001b).

#### 2.4.4 Independence at work

The INVRA‐Work questionnaire (Douma, Mulder, & Scholten, 2001a) is developed for people with intellectual disabilities to assess independence at work. It contains 38 items covering three domains: performance at work, motor competence and attitude at work. For each item, a score from 0 to 3 could be given which reflects to what extent something was performed independently, on the participant's own initiative, and in an acceptable manner. The sum of the three scales was used for the analyses. INVRA‐Work has a moderate inter‐rater reliability and a high internal consistency (Douma et al., 2001a).

#### 2.4.5 Support needs

To evaluate participants’ support needs, the SIS (Thompson et al., 2010) was used. The SIS is a semistructured interview developed by the American Association of Intellectual and Developmental Disabilities to assess the intensity of support someone requires to successfully perform several activities. Section 1 includes 49 activities grouped into six domains: home living, community living, lifelong learning, employment, health and safety and social activities. Section 2 consists of eight items addressing protection and advocacy. A trained interviewer collected the information from a participant's personal tutors. They had to answer whether the participant would require support when having to successfully perform a certain activity and if so, how frequent, how long and what kind of support would be needed. For the analyses, the total score of sections 1 and 2 was used, ranging from 0 to 655. Higher scores mean greater support needs. The reliability and validity were found to be sufficient to excellent (Buntinx, Maes, Claes, & Curfs, 2010; Claes, Van Hove, van Loon, Vandevelde, & Schalock, 2009).

#### 2.4.6 Psychopathological behaviour

The Adult Behavior Checklist (ABCL; Achenbach & Rescorla, 2003) was filled in by the participant's personal tutors to assess the occurrence of emotional and behavioural problems. In this questionnaire, 118 items are rated on a three‐point scale, indicating the frequency of occurrence of a particular type of behaviour (0 =  not at all, 1 =  a little or sometimes and 2 =  clearly or often). Items can be divided into eight syndrome scales: anxious/depressed, withdrawn, somatic complaints, Thought problems, Attention Problems, Aggressive Behaviour, Rule‐breaking behaviour and Intrusive behaviour. The first three syndrome scales form the Internalising Scale, and the latter three comprise the Externalising Scale. The total problem score consists of the sum of all 118 items. The mean scores for all scales were used for the analyses. The ABCL has shown to be a reliable and valid instrument to assess psychopathology in people with intellectual disabilities (Tenneij & Koot, 2007).

#### 2.4.7 Quality of life

Participants reported about their quality of life through five items from the World Health Organization Quality of Life Assessment‐5 (WHOQOL‐5; WHOQOL group, 1998a,b). The items encompass overall quality of life, and satisfaction with health, daily activities, relationships and living conditions. The validity and reliability of the WHOQOL‐5 were rated as acceptable to high, respectively (Geyh, Fellinghauer, Kirchberger, & Post, 2010). Questions were adapted to people with intellectual disabilities based on the WHOQOL for Disabilities (WHOQOL‐DIS; Power & Green, 2010). Questions could be answered on a scale from 1 to 4, with smiley faces (ranging from very unhappy to very happy) representing different levels of satisfaction. The total score was used for the analyses.

### 2.5 Statistical analyses

All data were analysed with IBM Statistical Package for the Social Sciences version 23.0. Descriptive statistics were used to summarize the scores of the participants on the various questionnaires. The scores of the questionnaires used for the analyses were normally distributed (*z*‐scores of skewness and kurtosis |<3.0|). No outliers (>3 *SD*) were found.

For the questionnaires, multilevel analyses were conducted to examine possible changes in participants’ functioning over time. In the first model, only a random intercept was included, and then the overall effect of time on the outcome measures was added as a linear effect. Next, in the final multilevel model, the present authors again used a random intercept and the separate measurement points were added as fixed factors. In this model, T1 (the start of participants’ AoI training) was used as a reference point, with which the scores on T0 and T2–T5 were compared. This enabled the analyses of whether any changes in functioning already occurred before the start of the training, as well as whether scores improved over time once the training had started. By using multilevel analyses and by collecting data at six different measurement points for each participant, the statistical analyses were less vulnerable to the limitations of a small sample size.

## 3 RESULTS

For all outcome measures, descriptive statistics per measurement point are presented in Table 2.

**Table 2:** Descriptive statistics for the scores on the questionnaires per measurement point (*n *=* *17)

	T0	T1	T2	T3	T4	T5
Independence						
SFSMR‐P^a^ mean item score, M (*SD*)	0.50 (0.17)	0.54 (0.14)	0.52 (0.11)	0.55 (0.12)	0.53 (0.12)	0.57 (0.11)
INVRA‐Home^b^ total score, M (*SD*)	251.20 (53.08)	277.65 (59.64)	–	266.19 (55.99)	–	285.13 (46.71)
INVRA‐Work^c^ total score, M (*SD*)	63.83 (19.80)	62.25 (15.72)	–	61.94 (18.08)	–	64.06 (14.15)
Support needs						
SIS^d^ section 1 + 2 total score, M (*SD*)	283.53 (56.68)	278.06 (51.70)	–	246.50 (69.27)	–	247.94 (45.33)
Psychopathological behaviour						
ABCL^e^ Internalising Scale mean item score, M (*SD)*	0.52 (0.31)	0.48 (0.31)	0.49 (0.22)	0.61 (0.35)	0.46 (0.24)	0.43 (0.21)
ABCL^e^ Externalising Scale mean item score, M (*SD*)	0.42 (0.33)	0.48 (0.32)	0.54 (0.38)	0.51 (0.33)	0.45 (0.28)	0.45 (0.25)
ABCL^e^ Total Problems Scale mean item score, M (*SD*)	0.41 (0.17)	0.48 (0.27)	0.51 (0.25)	0.54 (0.28)	0.46 (0.22)	0.45 (0.22)
Quality of life						
WHOQOL‐5‐DIS^f^ total score, M (*SD*)	15.29 (2.95)	15.65 (2.62)	15.53 (2.49)	15.20 (3.16)	16.50 (3.06)	15.63 (2.83)

*Notes*

a Social Functioning Scale for the Mentally Retarded‐Plus (range: 0–1).

b Inventory of Independence Aspects‐Home (range: 0–464).

c Inventory of Independence Aspects‐Work (range: 0–114).

d Supports Intensity Scale (range 0–655).

e Adult Behavior Checklist (range: 0–1).

f World Health Organization Quality of Life Assessment‐5 for Disabilities (range: 5–20).

### 3.1 Attainment of personal self‐management goals

**Figure 1:**
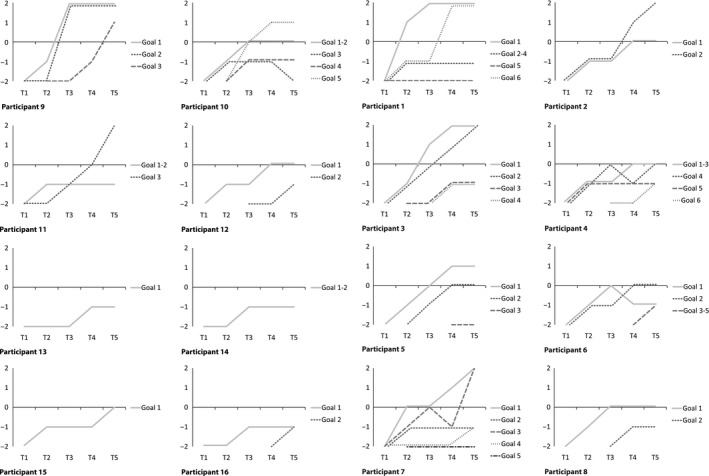
Goal attainment Scaling scores for each personal self‐management goal (PSMG) per measurement point (T1‐T5). Level 2: Participant has attained much more than the PSMG; Level 1: Participant has attained more than the PSMG; Level 0: Participant has attained the PSMG; Level −1: Participant has made progress, but not enough to attain the PSMG; Level −2: Participant's initial level before working on the PSMG

Figure 1 presents to what extent participants attained their PSMGs over time (except for the participant who dropped out). On average, participants worked on 3.4 PSMGs (range: 1–6 goals) during the 12 months the present authors followed them. Visual analysis of Figure 1 shows that all participants made progress in reaching one or more of their PSMGs, that is, for at least one PSMG they went from level −2 to level −1 or higher. Once a PSMG was attained (level 0), most participants kept working on other PSMGs, except for the person who dropped out, and for another person who left the training after obtaining his certificate after 10 months. Improvements regarding the attainment of PSMGs were mostly already made within the first 3 months. Initial achievements were generally maintained and additional improvements were continued to be made over time, both regarding the initial PSMGs and in some cases also regarding new PSMGs. Of the 52 PSMGs that were set in total, 26 PSMGs (50%) were attained by 13 participants taken together (level 0). For 13 of these 26 attained PSMGs (25% of the total), participants even exceeded the goal they had set (level +1 or +2). Regarding the 26 PSMGs that were not attained by 13 of the participants, mostly small improvements were nevertheless made (level −1). Only for four PSMGs no improvement occurred (level −2). However, in these cases the four corresponding participants attained at least one other PSMG. Furthermore, in three of these cases, participants started with this PSMG at a later stage (around T2, T3 or T4). One participant relapsed in a previously attained PSMG, although in the meantime she progressed in three other PSMGs.

### 3.2 Questionnaires

In the multilevel analyses of the various questionnaires, effects of age and gender were also explored, but no interaction effects were found with the various measurement points on any of the questionnaires (all *p*‐values >0.05).

### 3.3 Independence

The analysis of the SFSMR‐P data showed that there was no difference in the level of independence between T0 and T1. Furthermore, there were no differences between T2 and T5 compared to T1 (all *p*‐values > 0.05), indicating that the level of independence did not change once participants started with the training. Similar results were found for INVRA‐Home and INVRA‐Work.

### 3.4 Support needs

Participants’ support needs did not differ between T0 and T1. However, participants had significantly lower support needs at the following measurement points of the SIS, that is, T3 (*p *<* *0.01, *d *=* *0.57) and T5 (*p *<* 0*.01, *d *=* *0.55) compared to T1 (Table 3).

**Table 3:** Results of the longitudinal multilevel analysis of SIS scores with T1 as the reference point

	Coefficient (*b*)	*SE*	*t*	*F*	*p*‐value	95% confidence interval	
Intercept	278.06	13.20	21.07	443.99	0.00	251.08	305.04
T0	5.47	10.87	0.50	0.25	0.62	−16.37	27.31
T3	‐33.24	11.10	−2.99	8.97	0.00^a^	−55.55	−10.94
T5	‐31.80	11.10	−2.87	8.21	0.01^a^	−54.11	−9.50

*Notes*

SIS: supports Intensity Scale.

a *p *<* *0.01.

### 3.5 Psychopathological behaviour

The analysis of the ABCL Total Problems Scale revealed no significant differences in the occurrence of psychopathological behaviour when T0 was compared to T1, as well as when T2–T5 were compared to T1 (all *p*‐values > 0.05). Similar results were found when the Internalising and Externalising Scale were analysed separately.

### 3.6 Quality of life

There were no changes in quality of life, as measured by the WHOQOL‐5‐DIS. When comparing the scores at T0 to those at T1 and when comparing T2–T5 to T1, no significant differences were found (all *p*‐values > 0.05).

## 4 DISCUSSION

This study evaluated a self‐management training for people with intellectual disabilities that aims to enhance independent functioning in daily life and that can be tailored to individuals’ PSMGs, abilities and preferences regarding how they would like to work on these PSMGs. Results of the follow‐up measurements after the start of the training indicate that the training contributed to the attainment of PSMGs, while also decreasing participants’ support needs. The level of independence was not affected, nor was the occurrence of psychopathological behaviour and quality of life.

### 4.1 Principal findings

This study's findings support a lifelong learning for people with intellectual disabilities. Significant improvements were observed in the attainment of PSMGs already within the first 3 months after the start of the training, and more improvements were continued to be made during the following months. All participants made improvements in attaining their PSMGs. Half of all PSMGs were attained, and for a quarter of these attained PSMGs, participants even exceeded the goal they had set. Regarding the unattained PSMGs, smaller improvements were nonetheless made. Although the PSMGs were often very specific, reaching these goals can be of great personal significance to an individual. Learning to cycle to work or learning to use the Internet, as was the case for some of our participants, can greatly contribute to one's community participation (e.g., Chadwick, Quinn, & Fullwood, 2017; Wright & Wolery, 2011). The training's effectivity was further supported by the finding that participants’ support needs significantly decreased once they started with the training. Elements of the training's approach, that is, tailoring to the individual, involving the support network and assisting the transfer of learnt skills to daily life, seem to benefit people with intellectual disabilities. This has already been suggested in previous research (Cavkaytar et al., 2017; Douglas et al., 2015; Gilson et al., 2017; Goldschmidt & Song, 2017; Hale et al., 2011; Kuijken et al., 2016; Young et al., 2012); however, our study for the first time evaluated a self‐management training in which all these elements were combined and which showed that this is a promising approach.

The finding that the level of independence did not change might be explained by the fact that participants only worked on specific PSMGs. Although these were aimed at promoting independence, it is plausible that attaining only some specific PSMGs does not significantly improve overall independence. The used outcome measures may also not be sensitive enough to detect these small improvements. Furthermore, not all participants’ PSMGs were included in the independence questionnaires, so the successful attainment of a PSMG was not necessarily reflected in these questionnaires’ scores. In addition, given the overall learning deficit of people with intellectual disabilities (American Psychiatric Association, 2013), significant improvements in independence are perhaps also not to be expected within 12 months’ time. Future studies have to focus on more long‐term effects of these types of self‐management trainings on independence. Another finding was that the occurrence of psychopathological behaviour and quality of life did not change. As the training did not directly target these domains, improvements in these domains might only occur over longer periods of assessments and if more PSMGs are attained and the level of independence increases (Dollar et al., 2012; García‐Villamisar et al., 2013). Regarding quality of life, it must also be noted that the present authors used a brief questionnaire to minimize the burden on participants. Therefore, not all aspects of quality of life (Schalock, 2004; Schalock, Verdugo, Gomez, & Reinders, 2016) could be included and the questionnaire might not have been sensitive enough to detect changes over time.

### 4.2 Limitations and future research

An important limitation of the study concerns the relatively small sample size which mostly consisted of people with mild intellectual disabilities. Whether the results can be generalized to all levels of intellectual disabilities is therefore unknown. Another limitation is that the present authors were unable to conduct a randomized controlled trial, as the problems of people with intellectual disabilities can be so diverse and complex that forming an adequate control group is challenging. Furthermore, 8 months after the AoI was implemented, almost all participants moved to a different apartment or group home within the organization's compound. For several months, participants were unsure of the house in which they were going to live, whom their fellow residents would be and which staff members would be working in their homes. As this was very stressful to them, this might have negatively affected participants’ functioning, thereby potentially having confounded our results. Last, the support network was perhaps not always optimally involved. Although the present authors did not conduct a process evaluation, the support staff from other locations were possibly not always sufficiently aware of what participants were training at the AoI and how to support their development at home or at day care. This might have limited the transfer to daily life and the effects of the training. Such a process evaluation would have been of additional value to this study, just as the inclusion of interviews or focus groups with participants, trainers, support staff and family members about how they evaluated the training. This could have increased the insight into effects of the training that cannot be easily measured through questionnaires. Regarding the latter, the use of direct observations of participants’ level of self‐management, independence, support needs and behaviour would also have contributed to a more complete view of the training's effects.

These limitations call for a further study with a larger sample, including people with various levels of intellectual disabilities. In addition to the inclusion of qualitative and observational measures, the use of validated self‐reports regarding the measured domains should be considered, to further investigate the perspectives of participants themselves. Extra attention to the transfer of learnt skills to daily life should also be facilitated, by ensuring that family and support staff are actively involved.

## 5 CONCLUSIONS

To our best knowledge, this is the first study to report on a self‐management training for people with intellectual disabilities that is broadly applicable and adaptable to people's different goals, abilities and preferences regarding their way of training for their goals. The training was found to contribute to the attainment of PSMGs and to the reduction in support needs once participants started the training. This promising result justifies continued research on its implementation and further evaluation of the training's effects on specific subgroups of people intellectual disabilities to study who benefits most from the training. Further research and implementation may not only positively influence the lives of people with intellectual disabilities by helping them manage their affairs more independently, but may also reduce the burden on family and support staff because of participants’ decreased support needs.

## CONFLICT OF INTEREST

The authors declare that they have no conflict of interest.
